# Data-driven malaria prevalence prediction in large densely populated urban holoendemic sub-Saharan West Africa

**DOI:** 10.1038/s41598-020-72575-6

**Published:** 2020-09-28

**Authors:** Biobele J. Brown, Petru Manescu, Alexander A. Przybylski, Fabio Caccioli, Gbeminiyi Oyinloye, Muna Elmi, Michael J. Shaw, Vijay Pawar, Remy Claveau, John Shawe-Taylor, Mandayam A. Srinivasan, Nathaniel K. Afolabi, Geraint Rees, Adebola E. Orimadegun, Wasiu A. Ajetunmobi, Francis Akinkunmi, Olayinka Kowobari, Kikelomo Osinusi, Felix O. Akinbami, Samuel Omokhodion, Wuraola A. Shokunbi, Ikeoluwa Lagunju, Olugbemiro Sodeinde, Delmiro Fernandez-Reyes

**Affiliations:** 1 Department of Paediatrics, College of Medicine, University of Ibadan, University College Hospital, Ibadan, Nigeria; 2 Childhood Malaria Research Group, College of Medicine, University of Ibadan, University College Hospital, Ibadan, Nigeria; 3 African Computational Sciences Centre for Health and Development, University of Ibadan, Ibadan, Nigeria; 4 Department of Haematology, College of Medicine, University of Ibadan, University College Hospital, Ibadan, Nigeria; 5 Faculty of Life Sciences, University College London, Gower Street, London, WC1E 6BT UK; 6 Department of Computer Science, Faculty of Engineering Sciences, University College London, Gower Street, London, WC1E 6BT UK

**Keywords:** Infectious diseases, Malaria, Public health, Computer science

## Abstract

Over 200 million malaria cases globally lead to half-million deaths annually. The development of malaria prevalence prediction systems to support malaria care pathways has been hindered by lack of data, a tendency towards universal “monolithic” models (one-size-fits-all-regions) and a focus on long lead time predictions. Current systems do not provide short-term local predictions at an accuracy suitable for deployment in clinical practice. Here we show a data-driven approach that reliably produces one-month-ahead prevalence prediction within a densely populated all-year-round malaria metropolis of over 3.5 million inhabitants situated in Nigeria which has one of the largest global burdens of *P. falciparum* malaria. We estimate one-month-ahead prevalence in a unique 22-years prospective regional dataset of > 9 × 10^4^ participants attending our healthcare services. Our system agrees with both magnitude and direction of the prediction on validation data achieving MAE ≤ 6 × 10^–2^, MSE ≤ 7 × 10^–3^, PCC (median 0.63, IQR 0.3) and with more than 80% of estimates within a (+ 0.1 to − 0.05) error-tolerance range which is clinically relevant for decision-support in our holoendemic setting. Our data-driven approach could facilitate healthcare systems to harness their own data to support local malaria care pathways.

## Introduction

Human malaria caused by *Plasmodium falciparum* is a mosquito-borne infectious disease threatening the lives of millions of people around the world. The World Health Organization (WHO) estimates that there were 212 million malaria cases globally in 2017^1,2^, with 429,000 resulting in death. Of these, 90% of cases and 92% of deaths occurred in Africa, predominantly in sub-Saharan regions (with 76% and 75% of global cases and deaths occurring in only 13 countries)^1,2^. Around the world, children under 5 years-of-age are the most vulnerable, accounting for an estimated 70.6% of all malaria deaths in 2016^3^. While various control and preventative interventions have been implemented over time, malaria still poses one of the greatest threats to human life.

An important set of control measures are the surveillance and estimation of burden of disease, to allow for strategic planning of already scanty healthcare and public health resources across endemic regions. Although the transformation of malaria surveillance into a core intervention has been designated as one of the three pillars of the Global Technical Strategy for malaria 2016–2030 (GTS)^4^, current surveillance and predictive systems are inadequate at accurately capturing and estimating the extent of malaria, particularly in highly endemic countries^5^.

**Figure 1:**
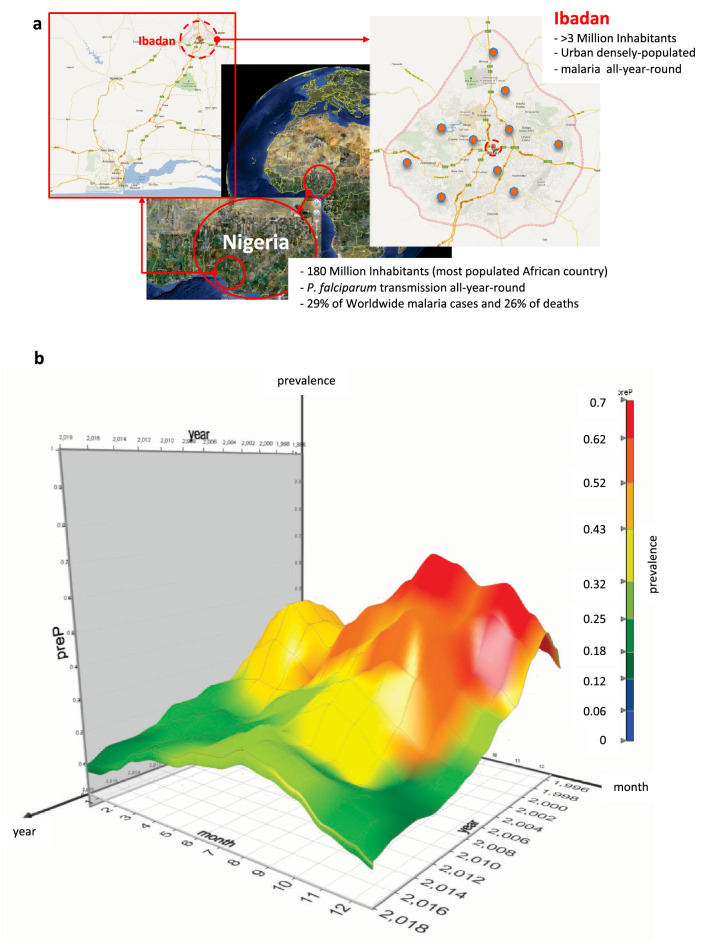
Study site geolocation and its monthly burden of malaria from 1996 to 2017. (**a**) Left and Centre: geographical location of the third largest urban large densely populated setting in Nigeria, the City of Ibadan. Right: Ibadan’s urban boundary; dropped-pin shows location of UCH Ibadan; red-balls shows location of primary and community centers. Images from Google Map data: Google, Maxar Technologies. By providing the previous attribution Google allows publishing of their images for non-commercial open access license as specified in their guidelines (https://www.google.com/permissions/geoguidelines/). (**b**) Ibadan dataset 3D surface-plot showing monthly mean malaria prevalence (y-axis and heat map); month (x-axis); year (z-axis) from 1996 to 2017.

The need for predictive systems that can reliably estimate future burden of malaria disease is particularly important for well-defined *Plasmodium falciparum* malaria in heavily affected countries such as Nigeria in sub-Saharan West Africa. In Nigeria, the most populous country of Africa with 180 million inhabitants, the entire population is at risk of malaria (i.e. no malaria-free areas), with 76% of the population living in all-year-round high-transmission areas^6^. Nigeria accounts for 29% of worldwide malaria cases and 26% of deaths in 2015 (mostly in children under five years of age), the largest proportion from any one country^7^. This global health challenge is particularly striking in large urban densely populated cities such as Lagos (> 15 million inhabitants) and Ibadan (> 3.5 million inhabitants) both under large all-year-round malaria burden where stretched healthcare resources will benefit from advance knowledge of malaria prevalence to support their specific malaria clinical care pathways (Fig. 1).

Malaria-estimation systems to date have employed classical mathematical-models of disease dynamics with varying degrees of success. Such models have been studied extensively, and historically have provided the foundations of reasoning about and formalizing the dynamics of several infectious diseases. They have been pivotal to formulation of transmission models aimed at understanding relationships between the malaria parasite, the host and the vector. More recently, model-based geo-statistics have provided important contributions to global estimates of the burden of malaria disease^8^. However, these approaches have been less effective in short-lead prevalence prediction in the context of region-relevant (local-scale) clinical pathways.

In contrast to classical models, data-driven supervised Machine Learning (ML) algorithms fit models to a given dataset with the key aim of extrapolating or predicting the future based on past observations, without the explicit incorporation of biological assumptions about the disease in question. This broad class of approaches are useful when the knowledge or concept about the application domain is poorly defined given its complexity such as in the case of malaria burden and disease dynamics. Machine learning approaches, as opposed to the explicit mathematical-model driven ones, offer a well-established set of data-harnessing algorithms that are well-suited for capturing complex data patterns from which to perform generalizable predictive tasks.

A 2012 scoping review on systems for predicting malaria burden of disease^9^ identified the use of mathematical modeling, regression, autoregressive integrated moving average and neural network approaches in 29 different studies spanning 13 countries. However, varying populations, sample sizes and non-openly available data sources made systematic comparison unfeasible. All studies differed in key aspects such as input features, prediction models, model evaluation measures and their performance. More recent studies have explored machine learning methods other than neural networks^10^, such as generalized linear models^11^, fuzzy association rule mining^12^, random forests^13^ and support vector machines^14–16^ with varying degrees of success and also using different and vastly heterogeneous non-openly available datasets (see Supplementary Table 1). Strikingly, none of the systems above have been derived from a care pathway support perspective, nor they have been deployed or are in clinical use. One recent study^17^ from a very-low seasonal non-holoendemic region reinforces the fact that although there have been enormous demands and efforts to develop predictive systems for malaria, no sustainable approach has been created. Altogether, this has translated to a lack of understanding concerning how regionally accurate short-lead predictions could influence the delivery of clinical malaria care pathways in resource constrained urban sub-Saharan healthcare systems. Moreover, our significant experience delivering high quality healthcare in a large urban holoendemic setting has provided us the insight that the usefulness of these systems rely not solely on accurate predictions, but also on empowering local healthcare providers to use their own data to produce predictions that can be acted upon within specific regional care pathways. Here, we present a solution that addresses both of these needs.

Apart from the modelling strategies used and lack of fine-grained openly available data, the development of malaria predictive systems^5,9,11,13–16^ has been severely hindered as collection of global data on malaria (vector, host, and environmental factors) is scanty, inaccurate and largely lacks quality control across all affected regions^5^. Only 10% of global malaria cases are reported through current systems^5^. This is also hindered by the challenges of access to reliable and accurate malaria diagnosis across malaria low-and-middle income holoendemic regions in the sub-Sahara. This in itself hinders the field, with an impossibility to test systems across regions.

In summary, a paucity of data, a tendency towards attempting universal models (one-fits-all-regions) and a focus on long-lead predictions have hindered development and deployment of regionally relevant systems. Here, we make a potentially important step forwards by overcoming these previous challenges. We provide a usable framework at the care center level, trained on more open data and with comparability of ML modeling strategies, that has direct potential for clinical translation.

To anticipate our findings, we designed, developed and validated a malaria prevalence predictive system using supervised machine learning on a unique 22-year large quality-controlled and prospectively collected malaria dataset that encapsulates a snapshot of the burden of *P. falciparum* malaria in the large densely-populated city of Ibadan, Nigeria. We show that this data-driven ML framework is able to extract complex patterns among features of this large malaria burden snapshot to reliably predict next-month malaria prevalence, which in these clinical settings is required to provide care pathway support. The proposed Region-specific Elastic-Net based Malaria Prediction System (REMPS) shows good generalization performance, both in magnitude and direction of the prediction, when tasked to predict short-lead next-month prevalence on previously unseen validation data. To the best of our knowledge, this work is the first to exploit the qualities of the elastic net to develop a simple and deployable malaria prediction system suitable for a high-transmission sub-Saharan holoendemic (all-year-round) setting where it is critical that the system falls within a small and usable error tolerance range.

## Methods and materials

### Ethics statement

The internationally recognized ethics committee at the Institute for Advanced Medical Research and Training (IMRAT) of the College of Medicine, University of Ibadan (COMUI) approved this research on the platform of the Childhood Malaria Research Group (CMRG) within the academic Department of Pediatrics, University of Ibadan, as well as at school and Primary Care centers throughout the city of Ibadan with permit number: UI/EC/10/0130. Parents and/or guardians of study participants gave informed written consent in accordance with the World Medical Association ethical principles for research involving human subjects.

### Study site

Data used in this study has been routinely prospectively collected by the Department of Pediatrics of the College of Medicine of the University of Ibadan (COMUI), University College Hospital (UCH), Ibadan, Nigeria located in sub-Saharan West Africa (Fig. 1a).

The city of Ibadan is a large urban metropolis with well over three million inhabitants, the third largest city in Nigeria, with all-year-round (holoendemic) malaria transmission^18^ (Fig. 1b). Urban Ibadan is one of the most densely populated areas in Nigeria. The city has a lengthy 8-month rainy season, with an average of 10 rainy days per month between May and October. Malaria transmission and clinical disease occurs throughout the year (Fig. 1b).

Our healthcare system at UCH-Ibadan is the largest and main academic system in urban Ibadan as well as the first teaching hospital in Nigeria. Our over seven-decades long experience on providing malaria clinical care makes our system a basin-of-attraction for healthcare from all regions of this large city with a catchment area of over three million inhabitants. Added to our all-clinical-services 800-bed tertiary-care system, we also provide all-specialties secondary-care as well as primary-and-community care services across the catchment area (Fig. 1a).

The city of Ibadan has both good road connectivity and wide access to transport to and from our healthcare settings across the city. Connectivity via a well-established but congested road network includes a variety of transport media with a wide range of affordability. Most individuals access our healthcare system via moto-taxis (cheap and avoids traffic congestion) which are ubiquitous across the metropolis. As malaria in Ibadan is truly holoendemic (Fig. 1) and our malaria clinical pathways have a track record on low mortality rates, we have a very active care-seeking behaviour to our services. Moreover, we also provide the best standard of malaria diagnostic services which make us the primary choice by large sectors of the population.

### Study design

We routinely screen for malaria and parasite-density using Giemsa blood thick and thin films all children up to 16 years-of-age attending any of our well-children or ill-children services. Our clinical services are: emergency ward; in-patient wards; out-patient clinics; routine school well-children malaria screening activities as well as secondary and primary care screening. Every year, we carry-out approximately 5 × 10^3^ malaria microscopy screens across all our clinical services listed above. The data used in this study includes all those screened in all our services from January 1996 to December 2017 inclusive, a total of 22 years (Tables 1, 2 and Supp. Table 2).

**Table 1:** Overall characteristics of training (DTRAS) and validation (DVALS) of Ibadan dataset.

Ibadan dataset	Number years	Dates	Months	*M* row-vector instances	*N* variables
Training set (DTRAS)	19	1996–2014	Jan–Dec	228	15
Validation set (DVALS)	3	2015–2017	Jan–Dec	36	15

**Table 2:** Ibadan dataset monthly aggregated variables.

Instance variables (*N* = 15)														
Index *x*	Variable name	Description	Units	Aggregate										
1	*Year*	Year $i$	$i$ = 1996–2017	No										
2	*Month*	Month $j$	$j$ = 1–12	No										
3	*Number-screened*	Total number screened	Integer	Sum $Month_{i,j}$										
4	*Median-age-neg*	Median age of malaria-negative	Age (months)	Median $Month_{i,j}$										
5	*Median-age-pos*	Median age of malaria-positive	Age (months)	Median $Month_{i,j}$										
6	*IQR-age-neg*	IQR age malaria-negative	Age (months)	IQR $Month_{i,j}$										
7	*IQR-age-pos*	IQR age malaria-positive	Age (months)	IQR $Month_{i,j}$										
8	*x-pd*	Mean of blood parasite densities^a^	MPs/μl	Mean $Month_{i,j}$										
9	*sd-pd*	STD of blood parasite densities^a^	MPs/μl	STD $Month_{i,j}$										
10	*mm-rf*	Month total rainfall	mm	Sum $Month_{i,j}$										
11	*mmP-rf*	Proportion of that year $i$ total rainfall	Proportion											
12	*Min-temp*	$Month_{i,j}$ minimum temperature	Celsius											
13	*Max-temp*	$Month_{i,j}$ maximum temperature	Celsius											
14	*x-temp*	Month mean temperature	Celsius	Mean $Month_{i,j}$										
15	*Prep*	$Month_{i,j}$ malaria prevalence^b^	Proportion											

Each row-vector d of D (index *x*)^c^														
1	2	3	4	5	6	7	8	9	10	11	12	13	14	15

*IQR* Inter-Quartile-Range, *STD* Standard Deviation.

^a^Parasite density (pd) = malaria parasites per microliter (MPs/μl) = (number-observed-malaria-parasites/number-observed White Blood Cells (WBC)) × 8000.

^b^Proportion of screened with confirmed malaria.

^c^Row-vector *d* of *D* (variable 1)–(variable 2) form the unique year-month key for that instance.

### Dataset characteristics

Demographics (year, month and age) and malaria clinical data (malaria diagnosis and parasite density) used in this study have been continuously collected between January 1996 and December 2017 as explained in the previous section. Overall demographic yearly aggregates are given in Supp. Table 2.

For this study, our Ibadan dataset was processed to consist of the monthly aggregated variables from larger datasets collected under our standardized routine malaria-screening which is linked to our clinical care pathways and departmental surveillance figures^18–24^ (Tables 1, 2 and Supp. Table 2). Our prospectively collected dataset is linked to and amalgamates our childhood malaria case–control and longitudinal studies and bio-banks^18–25^, as well as our research and development of an fast automated machine-learning-driven optical-malaria-diagnostic microscope^26^. The aggregated data used in this study is described in Tables 1, 2 and Supp. Tables 2 and 3. All data from our different clinical services are centralised in our malaria clinical pathways database ledger which is weekly processed to provide anonymised aggregates for the REMPS system. In the cases where an individual is tested several times within a day, we use the last test of that day to define her/his malaria parasite status. As malaria in Ibadan is truly holoendemic, we allow the aggregate script to count an instance every time that an individual is sampled for malaria parasites (either attending well-clinics, community sampling or attending to clinical services) except in the case of severe malaria in-patients that are counted only-once for the length of stay of that severe episode in the month that started. Readily usable aggregates are available at the end of month so short-lead prediction could be obtained.

We assembled our full Ibadan dataset, denoted by *D*, by aggregating data for each month from January 1996 to December 2017 (22 years), creating thus a total of 264 (22 × 12) entries (Table 1), each containing the following 15 variables (Table 2) namely: (1) year (not aggregated); (2) month (not aggregated); (3) total number screened (sum); (4) median age (months) of malaria-negative; (5) median age (months) of malaria-positive; (6) age (months) inter-quartile range of malaria-negative; (7) age (months) inter-quartile range of malaria-positive; (8) mean blood parasite density (MPs/μl); (9) standard deviation of blood parasite density (MPs/μl); (10) total rainfall (mm); (11) proportion of that year total rainfall; (12) minimum temperature (°C); (13) maximum temperature (°C); (14) mean temperature (°C) and (15) malaria prevalence (proportion of those who were screened and have confirmed malaria).

Our full Ibadan dataset is therefore a matrix *D* where each entry or row-instance of *D* is represented by a 1 by *N* = 15 vector *d* encoding the variables [*year*; *month*; *number-screened* = month total number screened for malaria parasites; *median-age-neg* = month median age of malaria parasite negative; *median-age-pos* = month median age of malaria parasite positive; *iqr-age-neg* = month interquartile range of malaria parasite negative; *iqr-age-pos* = month interquartile range of malaria parasite positive; *x-pd* = month mean malaria parasite density; *sd-pd* = month standard deviation of malaria parasite density, *mm-rf* = month total rainfall; *mmP-rf* = month proportion of that year total rainfall; *min-temp* = month minimum temperature, *max-temp* = month maximum temperature; *x-temp* = month mean temperature; *prep* = month malaria prevalence] (Tables 1 and 2).

### Malaria screening

Malaria parasites (MPs) were detected and counted by microscopy following Giemsa staining of thick and thin blood films^8–24,27^. The criterion for declaring a participant to be malaria parasite-free was no detectable parasites in 100 high-power (100×) fields in both thick and thin films. We validated the diagnosis outcome by randomly selecting one in ten thick blood films for independent review by local external experienced senior malaria-microscopy technologists. Parasite Density (PD), malaria parasites per microliter (MPs/μl), are calculated by dividing the number-of-observed MPs by the number-of-counted White Blood Cells (WBC) and then multiplied by 8 × 10^3^ as per widely established^18–24,27^.

### Environmental variables

All of Ibadan’s weather variables (rainfall, temperature) were acquired from the International Institute for Tropical Agriculture (IITA) Ibadan, Nigeria; (https://www.iita.org) that has kept Ibadan’s records since 1967.

### Dataset features and encoding of prediction tasks for supervised machine learning

The full Ibadan dataset *D* comprises of the two following datasets: (1) a Training Set (DTRAS) containing all the instances from the years 1996 to 2014 (19 years) as a *M* × *N* matrix where *M* = 19 × 12 = 228 row-vector instances and *N* = 15 variables (Tables 1 and 2) and; (2) a Validation Set (DVALS) containing all the instances from the years 2015 to 2017 (3 years) as a *M* by *N* matrix where *M* = 3 × 12 = 36 row-vector instances and *N* = 15 (Tables 1 and 2). The encoding of predictions task are described in detail in the “Supplementary Information” and Supp. Table 3.

### Supervised machine learning regression approaches

**Figure 2:**
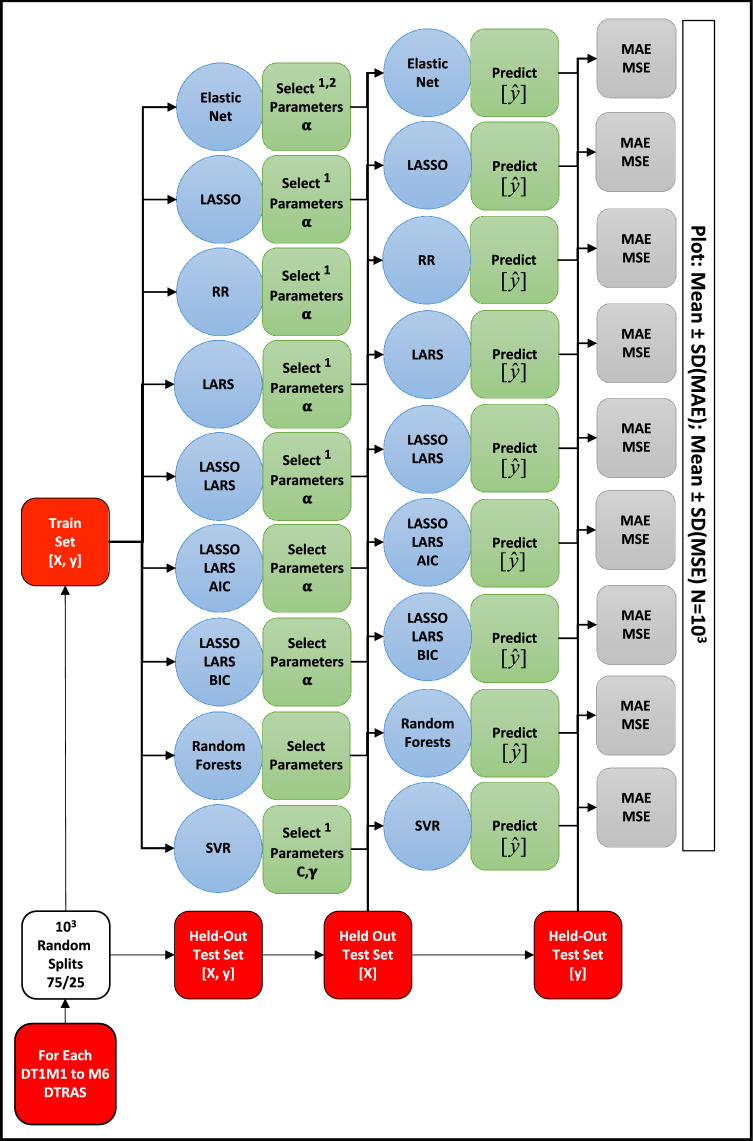
Machine learning algorithms parametrization, evaluation and model selection on the Ibadan training DTRAS dataset. DTRAS, Ibadan Dataset Training Set [from 1996 to 2014]; EN, elastic net; LASSO, least absolute shrinkage and selection operator; RR, ridge regression; LARS, least angle regression; AIC, akaike information criterion; BIC, Bayesian information criterion; SVR, support vector regression; $\alpha$, regularization strength parameter; C, SVR margin parameter; $\gamma$, SVR sigma gaussian-kernel parameter; MAE, mean absolute error; MSE, mean square error; X, features; y, true prevalence; $\hat{y}$, predicted prevalence. ^1^Using fivefold cross validation; ^2^L1Ratio = 0.5.

To build the predictive regression system we used Generalized Linear Models (GLM), Ensemble Methods (EM) and Support Vector Machines (SVM) within a supervised learning framework (Figs. 2 and 4) and explained in the next section. Technical details of these algorithms are presented in the “Supplementary Information”.

### Supervised learning algorithm parametrization, evaluation and model selection

For algorithm parametrization and evaluation, each of the training DTRAS datasets encoding the DT1M1 to M6 regression tasks, DT1M1-DTRAS to DT1M6-DTRAS (Supp. Table 2), were randomly split 10^3^ times into a Train Set (TS) containing 75% of the instances and a Held-Out Test Set (HOTest) containing 25% of the instances (Fig. 2). The TS is a *M* × *N* matrix where *M* = ceiling (0.75 × number-of-instances) and *N* = (number-of-variables per each T1M1 to M6 tasks) Supp. Table 3. The HOTest is a *M* × *N* matrix where *M* = (rest of the number-of-instances not in TS) and *N* = (number-of-variables per each T1M1 to M6 tasks).

**Figure 3:**
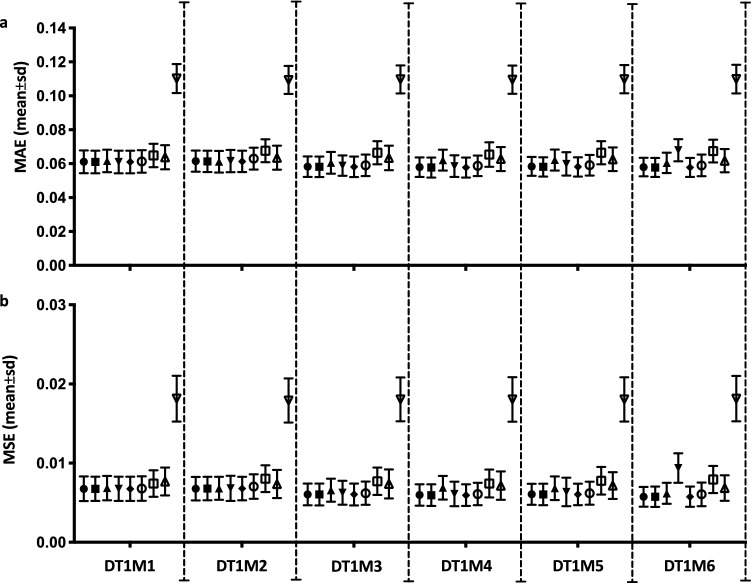
MAE and MSE errors of used machine learning approaches on training DTRAS dataset. (**a**) Mean and Standard Deviation MAE. (**b**) Mean and Standard Deviation MSE. Algorithms in order from left to right per each regression task DT1M1–DT1M2: EN (filled circles); LASSO (filled squares); RR (filled up-triangles); LASSO-LARS (filled down-triangles); LASSO-LARS-AIC (empty circles); LASSO-LARS-BIC (empty squares); RF (empty up-triangles) and SVR (empty down-triangles). *DTRAS* Ibadan Dataset Training Set [from 1996 to 2014], *EN* elastic net, *LASSO* least absolute shrinkage and selection operator, *RR* ridge regression, *LARS* LEAST ANGLE REGRESSION, *AIC* Akaike information criterion, *BIC* Bayesian information criterion, *SVR* support vector regression, *MAE* mean absolute error, *MSE* mean square error.

Each TS [X, y] was then used for the parameterization of each regression task algorithm within the framework (Fig. 2). For tuning the hyper-parameter alpha (regularization strength) for RR, a set of alphas = [1–3, 10, 102] were used and the best alpha selected by fivefold cross-validation on the TS (Fig. 2). For alpha selection in LASSO, EN, LARS, LASSO-LARS, we used model-specific iterative fitting along regularization path and selecting the best model by fivefold cross-validation on the TS (Fig. 2). Selection of best parameters was carried out using MSE as implemented in the scikit-learn Python library^28^. We parametrized the meta-estimator RF with number-of-trees = 10; maximum-features = number-of-features; nodes are expanded until all leaves are pure or until all leaves contain less than 2; using bootstrap when building trees (Fig. 2). For SVR we used a Gaussian kernel and carried out fivefold cross-validation to parametrize C and γ with the following grid search C = [1, 10, 10^2^, 10^3^, 10^4^] and γ = [1, 10^–1^, 10^–2^, 10^–3^, 10^–4^] respectively. After each parametrization, the algorithm was trained on the TS with the optimal parameters and predictions were made on the target outcome $\hat{y}$ (prevalence of following month) on the X instances of HOTest (Fig. 2). The trained algorithm test performance was then measured by MAE and MSE (Fig. 2) and mean ± SD of MAE and MSE over the 10^3^ random splits of DT1M1-DTRAS to DT1M6-DTRAS (Fig. 3).

### Error measures and parameter tuning

Mean absolute error (MAE), mean square error (MSE), Pearson correlation coefficient (PCC) measures were used when evaluating the quality of predictions of malaria prevalence (“Supplementary Methods”). For assessment on validation set see following section.

### L1–L2 ratio and regularization strength elastic net parametrization

**Figure 4:**
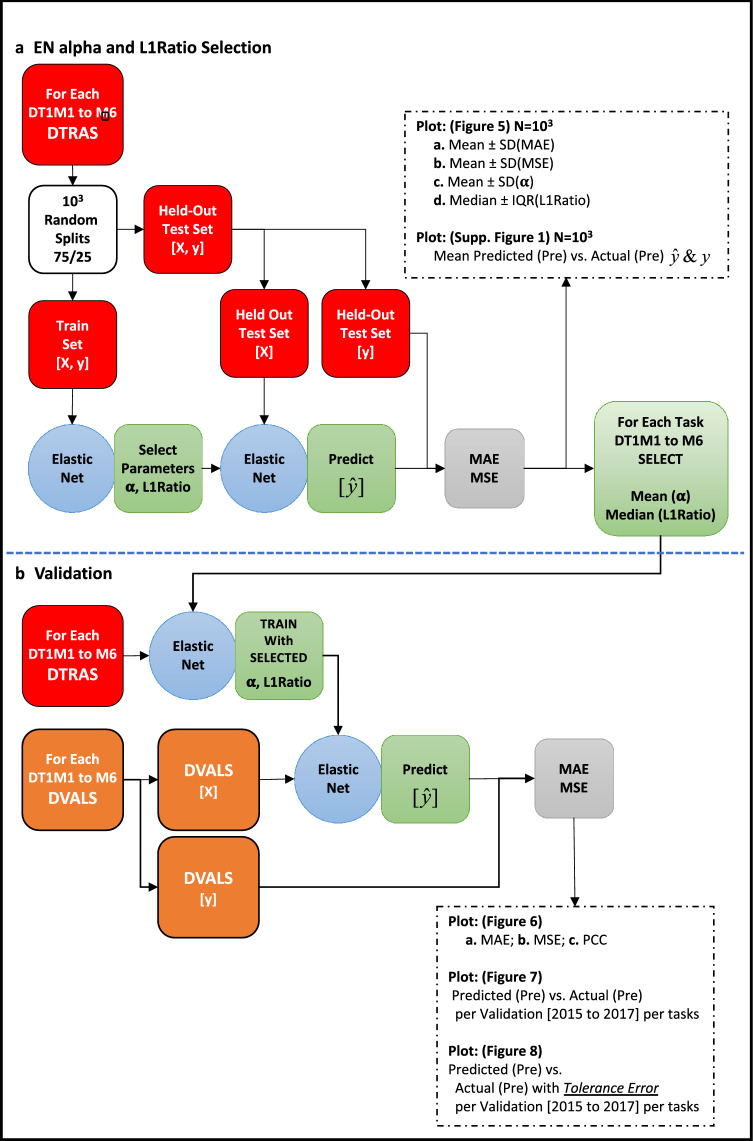
The Region-specific Elastic Net based Malaria Prevalence prediction System (REMPS). (**a**) REMPS regularization strength and L1-norm ratio model selection on training DTRAS dataset. (**b**) REMPS validation on DVALS dataset. DTRAS, Ibadan Dataset Training Set [from 1996 to 2014]; DVALS, Ibadan Dataset Validation Set [from 2015 to 2017]; $\alpha$, regularization strength parameter; MAE, mean absolute error; MSE, mean square error; X, features; y, true prevalence; $\hat{y}$, predicted prevalence. ^**1**^Using fivefold cross validation.

After selecting EN as the main ML algorithm for the system, we parametrized both $\alpha$ (regularization strength) and the L1-norm to L2-norm ratio (L1Ratio) as illustrated in Fig. 4a as follows. Each of the training DTRAS datasets encoding the DT1M1 to M6 regression tasks, DT1M1-DTRAS to DT1M6-DTRAS, were randomly split 10^3^ times into a Train Set (TS) containing 75% of the instances and a Held-Out Test Set (HOTest) containing 25% of the instances (Fig. 4a). The TS is a *M* × *N* matrix where *M* = ceiling (0.75 × number-of-instances) and *N* = (number-of-variables per each T1M1 to M6 tasks). The HOTest is a *M* × *N* matrix where *M* = (rest of the number-of-instances not in TS) and *N* = (number-of-variables per each T1M1 to M6 tasks).

**Figure 5:**
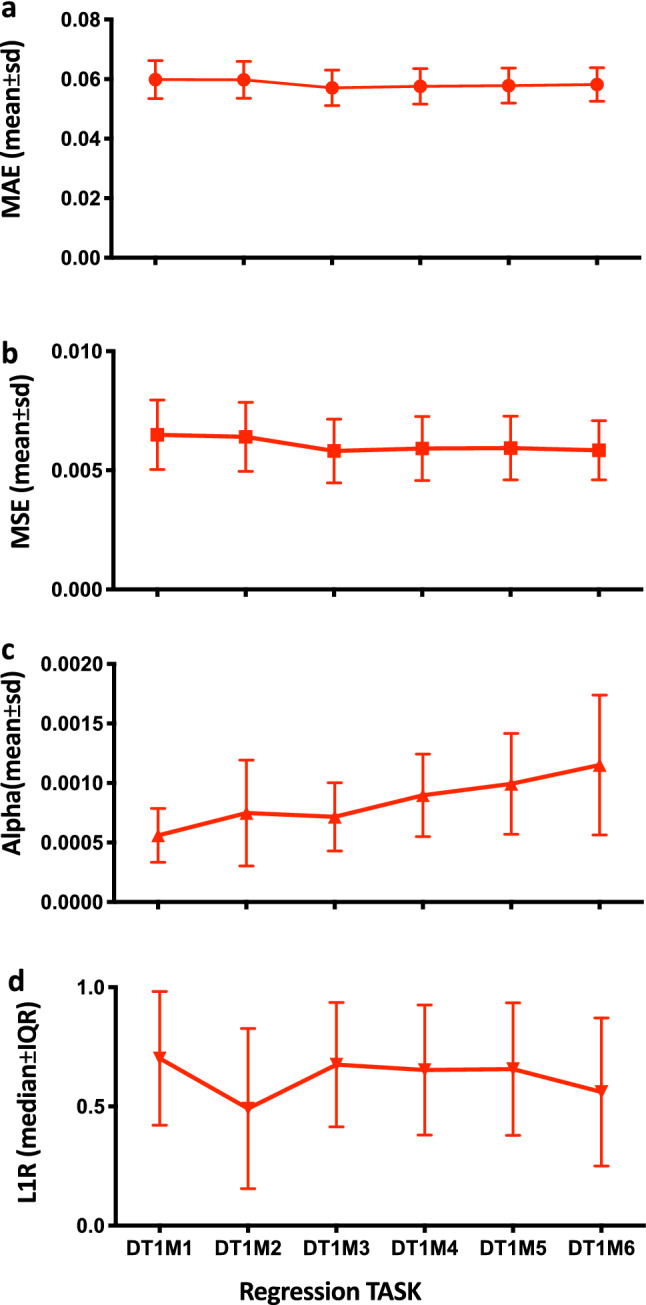
REMPS performance and best parameters range on training DTRAS dataset. (**a**) Mean and Standard Deviation MAE. (**b**) Mean and Standard Deviation MSE. (**c**) Mean and Standard Deviation of regularization strength parameter $\alpha$. (**d**) Median and Interquartile Range of L1/L2 norm ratio parameter L1Ratio. *DTRAS* Ibadan Dataset Training Set [from 1996 to 2014], *MAE* mean absolute error, *MSE* mean square error, *pre* prevalence.

**Figure 6:**
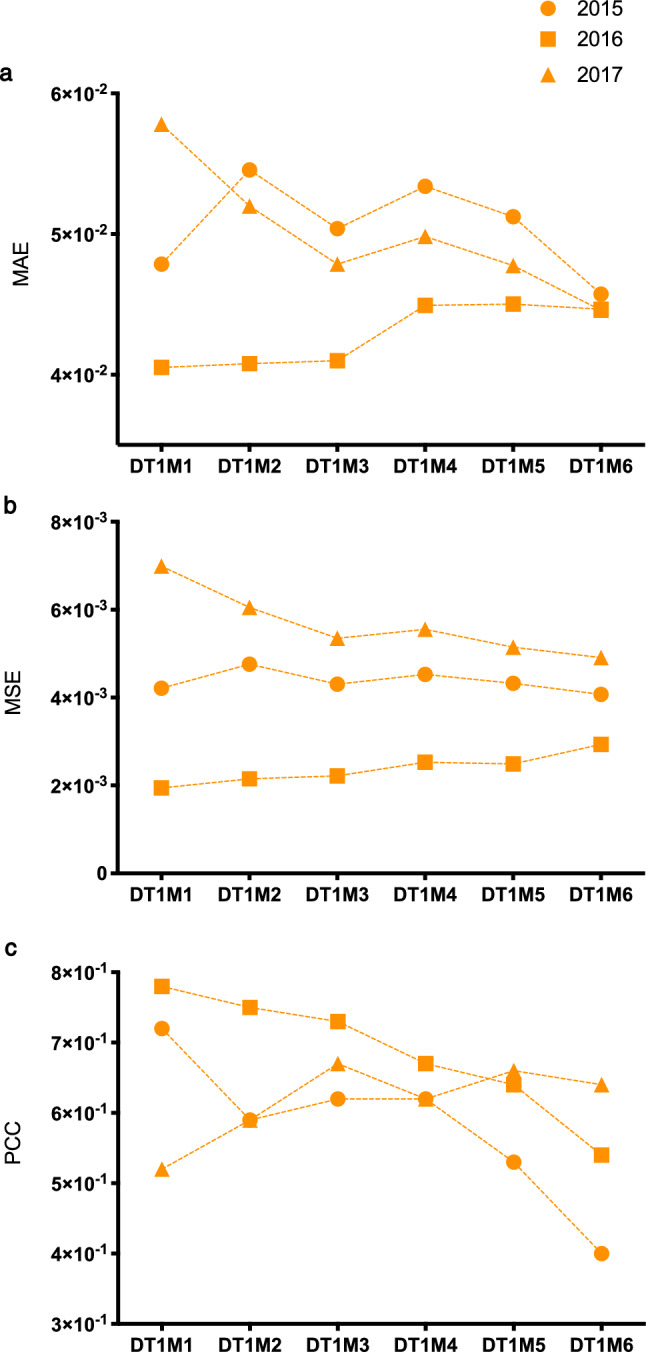
REMPS performance on validation set DVALS. Final REMPS system yearly MAE, MSE and PCC on 2015 (filled orange circles), 2016 (filled orange squares) and 2017 (filled orange triangles) DVALS validation set on all regression tasks DT1M1–DT1M6. *DVALS* Ibadan Dataset Validation Set [from 2015 to 2017], *MAE* mean absolute error, *MSE* mean square error, *PCC* Pearson correlation coefficient, *pre* prevalence.

For EN $\alpha$ and L1Ratio we used model-specific iterative fitting along regularization path and selecting the best model by fivefold cross-validation on the TS (Fig. 4a). Selection of best parameters was carried out using MSE as implemented in the scikit-learn Python library^28^. After each parametrization, the EN was trained on the TS with the best parameters and predictions for target outcome $\hat{y}$ (prevalence of following month) were made on the X instances of HOTest (Fig. 4a). The trained algorithm test performance was then measured by MAE and MSE (Fig. 4a). The mean ± SD of MAE, mean ± SD of MSE, mean ± SD of $\alpha$s and median ± IQR of L1Ratio were plotted over the 10^3^ random splits of DT1M1-DTRAS to DT1M6-DTRAS (Fig. 5). The $y_{i}$ (true prevalence value of all instances $i$) versus the mean of $\hat{y}_{i}$ (mean predicted prevalence value over the times the instance $i$ was included in the HOTest) is plotted in Fig. 6 for all regression tasks.

### Validation of the region-specific elastic-net based malaria prediction system (REMPS) framework

**Figure 7:**
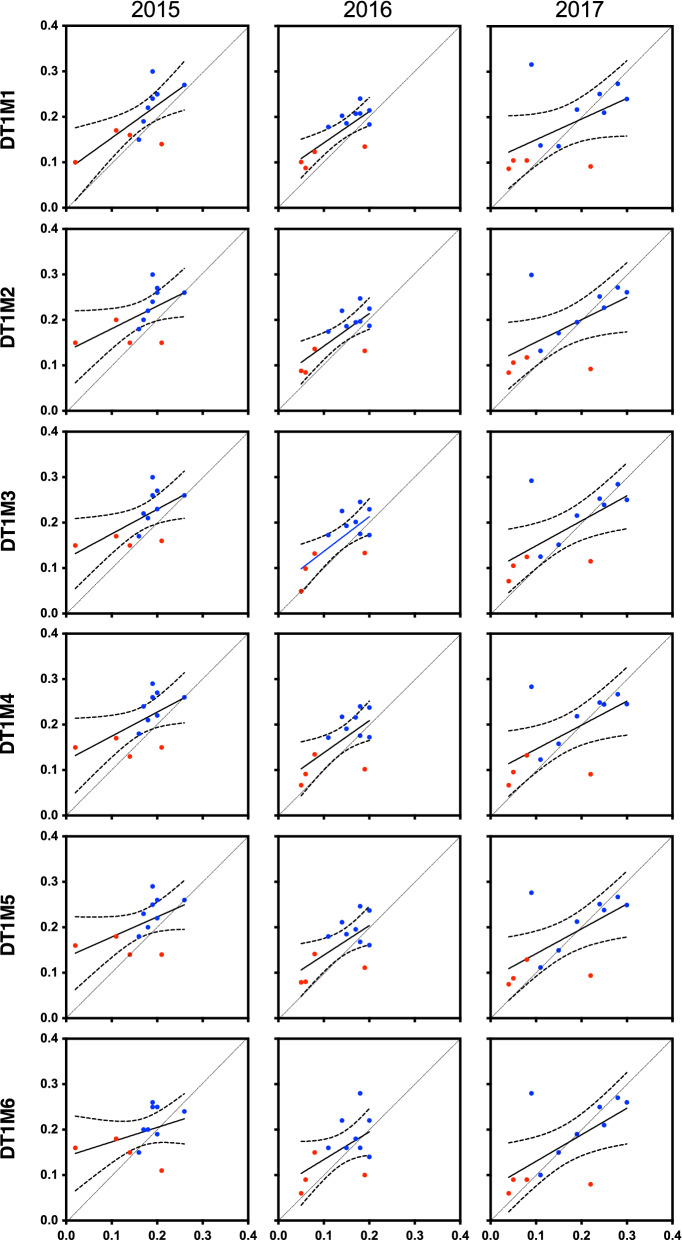
Scatter 2D plots of REMPS true and predicted prevalence on validation set DVALS. For all validation years 2015, 2016, 2017 and all regression tasks DT1M1–DT1M6. *x-axis*: true prevalence value $y$; *y-axis*: EN predicted prevalence value $\hat{y}$; red dots = dry season; blue dots = rainy season. *DVALS* Ibadan Dataset Validation Set [from 2015 to 2017]. Continuous black line = simple linear regression best fit line. Curved non-continuous lines = 95 CI of best fit line.

**Figure 8:**
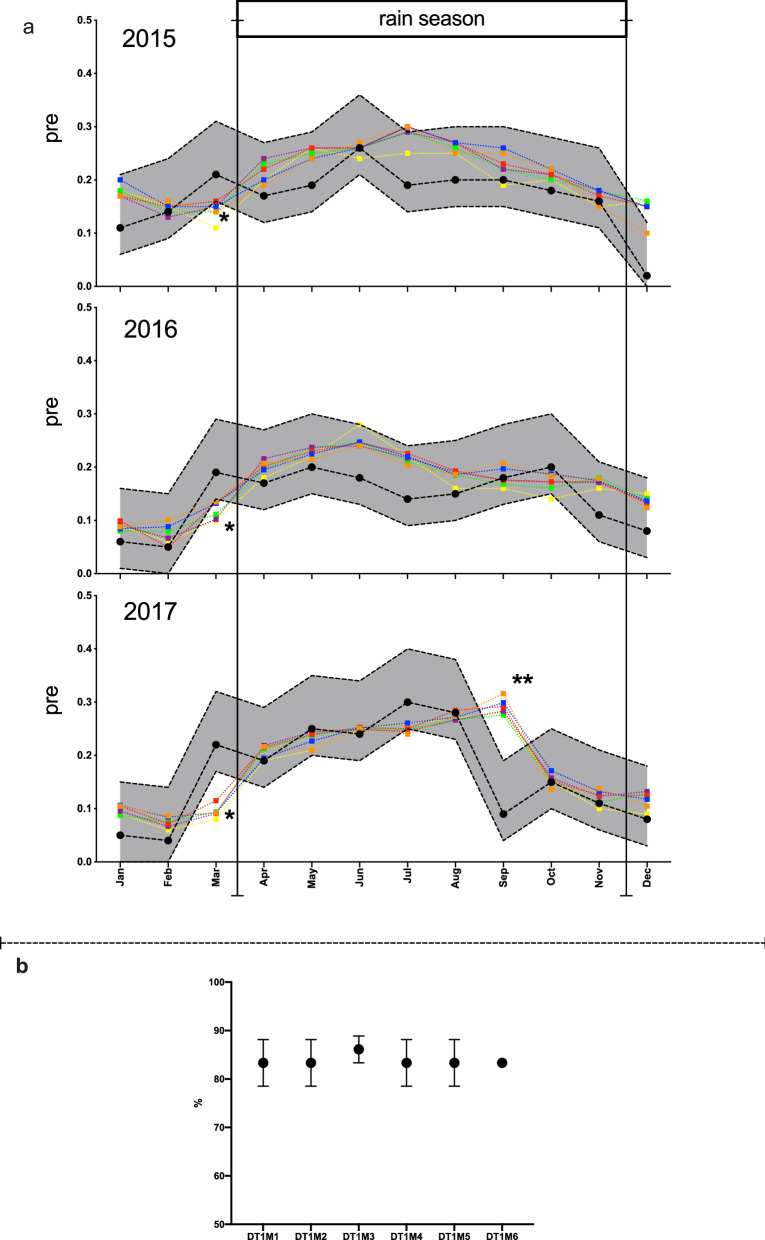
(**a**) REMPS predicted prevalence on validation set within regionally relevant tolerance-error. REMPS predicted prevalence for all validation years 2015, 2016, 2017 and all regression tasks DT1M1 to DT1M6 (orange, blue, red, purple, green, yellow filled squares respectively) plotted against the true prevalence value (black circles) and true prevalence value + 0.1 to − 0.05 tolerance-error (shaded grey area). (**b**) Mean REMPS prediction performance in % (y-axis) on validation set for each of the regression tasks DT1M1–DT1M6 (x-axis).

We show the validation of the trained EN on the regression tasks DT1M1-DTRAS to DT1M6-DTRAS datasets using respective validation datasets DT1M1-DVALS to DT1M6-DVALS (Figs. 4b, 6, 7 and 8) by using the best task specific $\alpha$ and L1Ratio parameters (Fig. 5c,d). Added to assessment of MSE (Fig. 6a), MAE (Fig. 6b) and Pearson Correlation Coefficient (Fig. 6c) we also assessed scatter plots of observed vs. REMPS predicted values (Fig. 7). Although widely used, current regression loss metrics (e.g. MAE, MSE, PCC, R2) have weakness on providing bounds of robustness which are exacerbated as dimensionality increases. We therefore used a problem domain context-relevant measure of how well the REMPS prediction falls within a clinically relevant range that allows the system to provide decision support in our holoendemic setting. In our settings the error-tolerance range of + 0.1 to − 0.05 is relevant and usable as a short-lead prediction (Fig. 8) to adapt our clinical pathways preparedness on a monthly basis.

## Results

### Study participants

We have coupled our clinical and community malaria screening services with data collection protocols and malaria diagnosis quality standards to ensemble a large and fine-grained dataset that encapsulates the burden of malaria disease within an urban densely-populated all-year-round high-malaria-transmission setting, the city of Ibadan, in the sub-Saharan West African region^18^ (Fig. 1a,b). The city of Ibadan is the third largest city in Nigeria, with over three million inhabitants. The city experiences a lengthy 8-month rainy season, with an average of 10 rainy days per month between May and October where malaria transmission and clinical disease occurs throughout the year (Fig. 1b).

In urban high-transmission holoendemic settings such as Ibadan, the burden of malaria vastly falls on children (Supp. Table 2). Although malaria predominantly affects children under 5 years-of-age, there is also a large burden in children up to 16 years-of-age. Therefore, we routinely screen for malaria all children attending any of our well-children or ill-children services across the city of Ibadan (Fig. 1a). Data used in this study comes from those screened in our services from January 1996 to December 2017 inclusive, a total of 22 years (Fig. 1b and Supp. Table 2). This Ibadan 22-years dataset is supported by the screening of > 9 × 10^4^ study participants (Supp. Table 2, Fig. 1b). Overall yearly aggregates of clinical demographics are described in Table 1. The Ibadan dataset *D* consists of a training set DTRAS with > 8 × 10^4^ subjects and a validation set DVALS with > 1 × 10^3^ subjects (Tables 1 and 2, Supp. Tables 2 and 3). Figure 1b shows that Ibadan’s malaria burden has decreased over the last 22 years. However, the city of > 3 million inhabitants (predominantly children) is still under a significantly large all-year-round burden of the disease, currently > 5% at its lowest during dry-season months of December and January.

### The elastic net consistently estimates with low error next-month prevalence across all regression tasks on training dataset

To select a supervised machine learning approach suitable for the task of predicting the malaria prevalence of the next month, we parametrized and trained nine algorithms [EN, LASSO, RR, LARS, LASSO-LARS, LASSO-LARS-AIC, LASSO-LARS-BIC, RF, SVR] (Fig. 2) on six regression tasks DT1M1 to DT1M6 (Supp. Table 3) using the DTRAS dataset (DT1M1-DTRAS to DT1M6-DTRAS) carrying out held-out test over 10^3^ random splits of the datasets (Fig. 2). The mean ± SD of MAE and mean ± SD MSE for each algorithm and for each regression task is shown in Fig. 3.

EN (Fig. 3, filled circles); LASSO (Fig. 3, filled squares); LASSO-LARS (Fig. 3, filled rhomboid) and LASSO-LARS-AIC (Fig. 3, empty circles), predictors performed consistently with low MAE (≤ 6.1 × 10^–2^) and low MSE (≤ 6.8 × 10^–3^) across all the regression tasks. RR predictors (Fig. 3, filled up-triangles) slightly decreased performance at regression tasks DT1M3 to DT1M6. LARS predictors (Fig. 3, filled down-triangles) were worst at the largest dimensionality of the task DT1M6. LASSO-LARS predictors using the BIC information measure (Fig. 3, empty squares) consistently performed worse than LASSO-LARS-AIC. RF predictors (Fig. 3, empty up-triangle), despite being consistent across tasks, performed slightly worse than EN, LASSO and RR. SVR predictors (Fig. 3, empty down-triangle) were also consistent across all regression tasks, but repeatedly had the worst performance when compared to all other algorithms on each of the tasks DT1M1 to DT1M2 with MAE (1.1 × 10^–1^) and MSE (1.8 × 10^–2^). For tasks DT1M1 and DT1M2, all predictors (except SVR) performed with MAE (≤ 6.8 × 10^–2^) and MSE (≤ 8 × 10^–3^) Fig. 3.

### Building a region-specific elastic-net based malaria prediction system (REMPS)

Using our Ibadan DTRAS dataset, we show that EN regularization-strength and L1-norm parametrization produce next-month prevalence estimates with low error and allows us to build a regionally adaptable Region-specific EN based Malaria Prediction System (REMPS).

We chose to concentrate our predictive model REMPS on the EN algorithm firstly because: (1) EN achieved consistently good performance across all DT1M1–DT1M6 (Fig. 3, Fig. 5) in the DTRAS data with MAE (≤ 6.1 × 10^–2^) and MSE (≤ 6.8 × 10^–3^) and (2) the L1-norm Ratio (that controls L1-norm vs L2-norm regularization) could be indeed useful in fine-tuning the system as the dynamics of the burden of disease change and/or it is used in another locality. For building REMPS, the EN was parametrized for [$\alpha$, L1Ratio] on the six regression tasks DT1M1 to DT1M6 using the DTRAS datasets (DT1M1-DTRAS to DT1M6-DTRAS) and carrying out held-out test over 10^3^ random splits of the datasets (Fig. 4a). The mean ± SD of MAE, mean ± SD MSE, mean ± SD of $\alpha$ and median ± IQR of L1Ratio for each regression task are shown in Fig. 5. L1Ratio tuned EN achieved similar consistent performance to those shown in the previous section with MAE ≤ 6 × 10^–2^ and MSE ≤ 6.5 × 10^–3^ (Figs. 3, 4a, 5a,b). For each task DT1M1 to DT1M6 the performance was achieved by a unique range of $\alpha$ and L1Ratios (Figs. 4a, 5c,d) and these parameters were used for building and validating the final REMPS system as described in the next section.

For each regression task DT1M1 to DT1M6 the $y$ (true prevalence value) and the mean $\hat{y}$ (predicted prevalence value) over 10^3^ random splits of DTRAS was plotted (Supplementary Fig. 1a–f dotted-black line and red line respectively). Over 10^3^ random splits of DTRAS each instance fell into the HOTest between 225 and 285 times. The mean of an instance predicted prevalence value, over the times that instance fell into the HOTest shows an overall good alignment with the true prevalence value of such instance across all tasks (Supp. Fig. 1a–f). This is consistent with the low MAE and MSE values observed (Supp. Fig. 1a,b). However, there are small subsets of instances that carry most of the error as follows: (1) labeled as (1) in Supp. Fig. 1a–f, from the 1996 to the 2000 period rain-season where the trained REMPS, despite agreeing with the direction, underpredicted prevalence; (2) labelled as (2) in Supp. Fig. 1a–f, during 1996 period dry-season, despite agreeing with the direction, the trained REMPS underpredicted prevalence and; (3) labelled as (3) in Supp. Fig. 1a,b (only DT1M1 and DT1M2), during the 2011 dry-season period, the trained REMPS did not agree with the direction and overpredicted prevalence.

### Validating the locality-specific elastic-net based malaria prediction system (REMPS)

The Elastic Net based system trained on best hyper-parameters estimates next-month prevalence with low error across all regression tasks on the 2015, 2016 and 2017 validation datasets. For each task DT1M1–DT1M6 the performance was achieved by a unique range of $\alpha$ and L1Ratios (Fig. 5c,d), information that was then used for building and validating the final system (Fig. 4b). The REMPS mean of $\alpha$ and the median of L1Ratio values obtained in the previous section (Figs. 4a, 5c,d) were chosen to build and validate the final REMPS system on a previously unseen set of instances from the 2015, 2016 and 2017 period, the DVALS dataset, as shown in Fig. 4b. For each DT1M1 to DT1M6 task an EN was trained using the full DTRAS dataset with selected parameters and its monthly performance was assessed on the 2015, 2016 and 2017 DVALS (Figs. 6, 7 and 8). On all regression tasks, the REMPS monthly prevalence predictions achieved consistently low MAE (≤ 6 × 10^–2^), low MSE (≤ 7 × 10^–3^) with Pearson Correlation Coefficients (PCC) ranging between 0.4 and 0.8 (Fig. 6).

To assess the quality and direction of these monthly validation predictions, a scatter 2D plot of predicted prevalence value versus true prevalence value for all DT1M1–DT1M6 prediction tasks is shown in Fig. 7 where red and blue dots represent rainy and dry season months respectively. The plots highlight the importance of interpreting the validation of the predictions in relation to the problem domain. For example, validation year 2017 shows very good prediction agreement (i.e. dots closer to the diagonal) except for two months (one rainy season and one dry season) which impairs its overall yearly PCC (Fig. 6). Therefore, to further evaluate these predictions within an error-tolerance which is relevant for making the system suited and usable for a high-transmission holoendemic setting, we plotted the predicted monthly prevalence for all tasks against the true prevalence with a + 0.1 to − 0.05 tolerance error (Fig. 8a). Overall, across all 216 monthly predictions on the 2015 to 2017 validation set (3 years × 12 months × 6 tasks), 80% were within the tolerance error + 0.1 to − 0.05 (Fig. 8b) which is operationally relevant for this holoendemic region and makes our system extremely usable for decision support in the Ibadan setting. During the long Ibadan rainy season (April–November), the REMPS is extremely robust (95% of predictions within range) in estimating monthly prevalence within the error-tolerance range (Fig. 8a), except during the month of September 2017 where extreme prediction outliers (Fig. 8a see**) made us suspect a critical event. We discovered that during that month, a country-wide general Nigerian Federal Government healthcare system strike had a nation-wide effect on our clinics. This reinforces the usefulness of our proposed system as a novelty detection system as in years 2015 and 2016 the REMPS was robust in estimating September’s month prevalence (Fig. 8a). During the dry season (December–March) the system also performs consistently within the error tolerance boundaries during the months December to February. However, during the month of March, for all validation years some prediction tasks underpredicted below the − 0.05 range, an effect that is most extreme on 2017 prediction (Fig. 8a see*). The month of March is the transition boundary from the dry to the rainy season and despite the trained REMPS mostly agreeing with the direction of the prediction, the magnitude of the estimates for year 2017 were on the − 0.1 range instead of − 0.05. We could not find a critical event explanation for such observation.

Although REMPS good generalization performance and low dimensionality of our dataset does not necessarily require us to adopt a feature selection strategy, we nevertheless explored how the system performed in those scenarios where a regional healthcare centre does not have records of the actual parasitaemia (Supp. Fig. 2). Despite harnessing the standard of malaria care features (age, sex, malaria diagnosis by gold standard microscopy and parasitaemia) plus readily available environment variables, parasite density is the least available feature variable of them all. We observed that the newly trained REMPS can indeed provide estimates within a range that can provide useful information for regional decision support (Supp. Fig. 2).

### Use cases and deployment analysis of locality-specific elastic-net based malaria prediction system (REMPS)

The REMPS system is easily deployable using current off-the-shelf hardware and thus opens the door to sustainable digital global health. The system could be further trained, deployed and developed using free open-source Python and ML tools provided within the freely available Anaconda Navigator environment. We propose a use case where each regional health center is a regionally trained EN node (harnessing such local data at its best) within an interconnected network of EN predictors, via a distributed ledger, where new nodes could use closer regional predictors while they refine their own predictors (Supp. Fig. 3).

The simplicity of REMPS provides an incentive for sub-Saharan centers by giving decision-support value to their own routinely collected malaria data. This in turn should encourage those centers to transfer such data (15 variables in this study) into simple digital format that can be exploited by themselves and by the network of REMPS predictors (Sup. Fig. 3). As the network of locally specialized REMPS predictors grows, it opens the possibility of meta-learning and novelty detection algorithms to be applied for tasks such as early epidemic prediction and more efficient distribution of resources across malaria affecting regions.

## Discussion

We have designed, developed and validated a machine-learning based system that is able to reliably predict next month malaria prevalence within urban densely-populated holoendemic malaria Ibadan with low error. The Region-specific Elastic Net based Malaria Prediction System (REMPS) shows good generalization performance, both in magnitude and direction of the prediction, when tasked to predict monthly prevalence of previously unseen data from years 2015, 2016 and 2017.

To the best of our knowledge, our work is the first to exploit the tradeoff qualities of the EN to predict malaria prevalence one-month ahead (short-lead time forecast) in an all-year-round malaria urban setting. Previous malaria studies in different world regions (summarized in Supplementary Table 1) and our 60-years Ibadan academic healthcare system knowledge formed the basis to select the variables incorporated into REMPS. Our system exploits 19 years [1996–2014] of host information (age, malaria status, parasite densities); temporal information (year, month) and; environmental information (rainfall, temperature), from a predominantly Yoruba, largely populated well-defined spatial urban setting living under high all-year-round malaria burden. Apart from environmental variables, the host variables used are derived from the gold-standard of malaria clinical care that has been in place for decades.

We used our region-specific data, the Ibadan dataset, to train a relevant REMPS which currently contributes to decision making on managing our clinical site malaria healthcare and surveillance resources. The trained REMPS has an error-tolerance within + 0.1 to − 0.05 across all prediction tasks which is appropriate for a system to be usable in the high-transmission holoendemic setting of Ibadan. The qualities of the ML approach include its simplicity and performance. Moreover, the low dimensionality (small number of variables) of the proposed feature dataset suggests that a feature selection strategy is not critical or desirable. While in classical modelling there is a tendency to remove variables from a model to assess their relative importance, the variables used by our system are established as part of the complex dynamics of the malaria lifecycle. The machine learning methods used are well theoretically suited to deal with the low dimensionality of the system proposed. We nevertheless present how the system performs in those scenarios where a regional healthcare centre does not have records of the host parasitaemia. We therefore propose to move away from a one-fit-all-regions approach where the EN is an excellent and simple enough tool, with its L1/L2 ratio trade-off, to allow to find a predictor customised for other regions such as catchment areas of healthcare centres in nearby Lagos. What we show with the performance of the REMPS with or without host-parameters that parametrising L1/L2 will result in a usable system that can handle local characteristics were these be on what data is available and/or their dynamics. Unfortunately, the diversity and lack of open data from previously published studies makes it hard to test our proposed approach in those previously published settings. However, our knowledge of the region makes us confident to expect that REMPS open availability and simplicity of deployment, retraining and parametrisation of our system will encourage sub-Saharan care centres to capitalise on their routinely collected data to inform their pathways.

During the long Ibadan rainy season (April–November) the system is extremely robust in estimating monthly prevalence within the error-tolerance range. The system has also shown novelty-detection capabilities by highlighting prediction outliers observed in collection of validation data from September 2017 which was affected by a personnel strike in the healthcare system. Interestingly, the system has shown the complexity of the dynamics of the burden of disease near the dry-to-rainy season transition period (i.e. March). This may be due to emerging patterns across this seasonal transition period as we have observed recent dramatic changes of environmental factors in the city of Ibadan. Furthermore, recent investment on Ibadan’s infrastructure may be playing a role in these changes. We expect that feature enrichment refinements focused on transition periods will allow the system to further improve its accuracy. These adjustments will have to take into account that, despite Ibadan’s malaria burden decreasing over the last 22 years, the city is still under a currently changing but still significantly large all-year-round burden of the disease (Fig. 1b).

We have shown that a data-driven machine learning approach offers an alternative that allows predictive systems to be created that do not rely on an explicit formulation of the disease process. We focused our system on the Elastic Net, as it produced stable results across all prediction tasks while also providing flexibility of tuning regularization strength as well as the L1- to L2-norm ratio. The EN is well suited for problems such as malaria prediction, where there are multiple features which are correlated with one another, and trading-off between L1-norm (LASSO) and L2-norm (RR) allows the system to retain stability. We show that the EN based system provides an efficient, yet flexible, system for all the regression tasks relevant to the clinical and epidemiological context within the region.

Previous ML systems^11,13–16^ have used significantly smaller datasets and none have harnessed features such as host-age and host-parasite-density. These host-features are thought to provide information on the not yet-understood complex relationships between host-immunity, host-genetics, parasite load and transmission burden. If these variables are available, our system can indeed allow a regional health center to harness such information. If not, we have shown that a REMPS can still perform within the proposed parameters for this holoendemic high-transmission setting and therefore enables these centers to benefit from our easily deployable approach while a distributed-ledger network of regional-predictor-experts can open the door to other machine learning approaches such as transfer learning to further assist in multiscale surveillance.

Historically the tendency has been to build monolithic predictive systems, despite malaria data being scanty and unprecise, that have been unable to provide accurate performance across different malaria regions. These monolithic systems cannot fairly be tasked with predicting good local estimates of prevalence while at the same time being able to accurately detect extreme pattern-changes globally. On the contrary, our results show the feasibility of a data-driven region-specialized malaria prevalence predicting system for a large metropolis of 3 million inhabitants in sub-Saharan West Africa. Our system can be used as a starting point to support the deployment of regionally specific systems across malaria affected regions such as the densely populated metropolis of Lagos and Kano in Nigeria. At our tertiary level, REMPS supports the readiness of our blood bank to sustain the near-zero mortality of our severe malarial anemia care pathways. At our primary community and peri-urban level, REMPS supports readiness for diagnosis and treatment of uncomplicated malaria. Our REMPS could be fine-tuned to support regionally dependent adaptability and readiness of healthcare pathways, each with their own critical bottlenecks, which is well recognised by the WHO as key for the global technical strategy for malaria. In rural settings, REMPS could facilitate the use of regionally specific data to tackle their own critical bottlenecks as well as allowing the interaction with urban settings to achieve this. In this context, our REMPS is a realisable step towards achieving truly data-driven open and distributed digital global health.

In view of the complexities faced with one-fit-all-regions explicit models, more emphasis could be placed on building meaningful multivariate data-driven region-specific systems designed to harness local data as the one presented in this study. Machine learning meta-models that take input from these regionally specialized systems could be most suited to provide vast regional epidemiological decision-support. We propose a deployment scenario where many regional centers, each a regionally trained REMPS node (harnessing such local data at its best), push their data and predictions into a distributed ledger that ensures consensus, consistency and immutability of information across participating nodes. New REMPS nodes could use closer regional predictors while they gear up to produce their own refined predictors. As the network of locally specialized predictors grows, it opens the possibility of meta-learning and novelty detection algorithms to be applied for tasks such as early epidemic prediction. Our system provides a step towards supporting efficient distribution of resources that takes into account the different locality-specific characteristics of malaria affected regions. Equally important, such a distributed ledger should provide an interface by which global healthcare authorities, policy makers and malaria control programs interact and support their decisions with regionally relevant data.

Finally, our validated REMPS system shows that local good-quality malaria longitudinal-data can be harnessed by current data-driven machine learning approaches to deliver locality-relevant predictions on burden of malaria. Reliable and adaptable malaria prediction systems can play key roles when deployed within a well-defined resource-stretched healthcare network as in the case of the large Ibadan metropolis where our system is deployed. In our large urban population settings, the system provides relevant short-lead next month prevalence estimates that are used for aiding decision making on critical aspects of urban to peri-urban care pathways. The deployment simplicity of our REMPS provides an incentive for other sub-Saharan centers, by enabling decision-support using their own routinely collected malaria data, to consider sustainable digital global health approaches to tackle challenges on healthcare provision in the region.

## Supplementary information

Supplementary Information.

## Data Availability

The dataset and code used in this study are openly available upon publication for ten years at the UCL open data platform following this link 10.5522/04/12369137. Links to REMPS data and code are also openly available with the open-access publication via (1) COMUI website; (2) by emailing the Childhood Malaria Research Group (CMRG), Department of Paediatrics, College of Medicine of University of Ibadan, University College Hospital, Ibadan, Nigeria. Emails: CMRG-Nigeria (paedcomui@yahoo.com) or; (3) by emailing the corresponding author (delmiro.fernandez-reyes@ucl.ac.uk).
