# Supplementary material for: Data-driven malaria prevalence prediction in large densely populated urban holoendemic sub-Saharan West Africa
Source: Sci Rep. 2020 Sep 28;10:15918. doi: 10.1038/s41598-020-72575-6 (PMC7522256; doi:10.1038/s41598-020-72575-6)
Supplement: Supplementary file 1 — Supplementary Information. [file 41598_2020_72575_MOESM1_ESM.docx]

**Data-Driven Malaria Prevalence Prediction in Large Densely Populated Urban Holoendemic sub-Saharan West Africa.**

Biobele J. Brown**^1,2,3^**, Petru Manescu**^3,6^**, Alexander A. Przybylski**^6^**, Fabio Caccioli**^6^**, Gbeminiyi Oyinloye**^1,2^**, Muna Elmi**^6^**, Michael J. Shaw**^6^**, Vijay Pawar**^6^**, Remy Claveau**^6^**, John Shawe-Taylor**^6^**, Mandayam A. Srinivasan**^6^**, Nathaniel K. Afolabi**^1,2^**, Geraint Rees**^5^**, Adebola E. Orimadegun**^1^**, Wasiu A. Ajetunmobi**^1^**, Francis Akinkunmi**^1^**, Olayinka Kowobari**^1^**, Kikelomo Osinusi**^1^**, Felix O. Akinbami**^1^**, Samuel Omokhodion**^1^**, Wuraola A. Shokunbi**^4^**, Ikeoluwa Lagunju**^1,2,3^**, Olugbemiro Sodeinde**^1,2,3,6^** and Delmiro Fernandez-Reyes**^1,2,3,6*^**

1. Department of Paediatrics, College of Medicine University of Ibadan, University College Hospital, Ibadan, Nigeria.
2. Childhood Malaria Research Group, College of Medicine University of Ibadan, University College Hospital, Ibadan, Nigeria.
3. African Computational Sciences Centre for Health and Development, University of Ibadan, Nigeria.
4. Department of Haematology, College of Medicine University of Ibadan, University College Hospital, Ibadan, Nigeria.
5. Faculty of Life Sciences, University College London, Gower Street, London, WC1E 6BT, United Kingdom
6. Department of Computer Science, Faculty of Engineering Sciences, University College London, Gower Street, London, WC1E 6BT, United Kingdom.

*****Corresponding Senior Author: Professor Delmiro Fernandez-Reyes. Department of Computer Science, University College London, Gower Street, London, WC1E 6BT, United Kingdom.

Email: Delmiro.Fernandez-Reyes@ucl.ac.uk

**This PDF file includes:**

Supplementary Text

Supplementary Figures S1 to S3

Supplementary Tables S1 to S3

SI References

Supplementary Information Methods

Dataset Features and Encoding of Prediction Tasks for Supervised Machine Learning

The full Ibadan dataset *D* comprises of the two following datasets: 1) A Training Set (DTRAS) containing all the instances from the years 1996 to 2014 (19 years) as a *M* by *N* matrix where *M* = 19 x 12 = 228 row-vector instances and *N* = 15 variables (Tables 1 and 1) and; 2) A Validation Set (DVALS) containing all the instances from the years 2015 to 2017 (3 years) as a *M* by *N* matrix where *M* = 3 x 12 = 36 row-vector instances and *N* = 15 (Tables 1 and 2). The encoding of predictions task is described in detail in the Supplementary Information.

Supervised trained ML models aim to predict a target outcome from a set of new unseen input variables (commonly referred to as features). The goal is to train a predictive system that, given the aggregated variables $\hat{X}$ collected at the end of the Month M1, can reliably estimate the malaria prevalence of the following Month (Task+1) $\hat{y}$ which is our target outcome. We therefore encoded the datasets DTRAS and DVALS as a supervised learning regression task. The task is denoted by the encoded Dataset Task+1 M1 (DT1M1) (Table 4) such that the relationship between the features $X$, a row-vector containing the aggregated variables for one month, and the outputs $y$, the prevalence observed the following month, can be learned by training a supervised ML model (Table 2 and Supplementary Table 2).

DT1M1-DTRAS (Table 4) is a *M*=228 by *N*=16 matrix where each row-vector instance $d_{i,j}=DTRAS(X_{i,j},y_{i,j+1})$ where *i*=year (from 1996 to 2014), *j*=month (January to December), $X_{i,j}$ is a 1 by *N*=15 row-vector containing all the variables for that month *j* of the year *i* (including the prevalence value of that unique month) and $y_{i+,j+}$ ,with $\left\{\begin{matrix} i_{+}=i+(j+1)/12 \\ j_{+}=(j+1)/12 \end{matrix}\right.$, is the malaria prevalence target value of the following next month. Similarly, DT1M1-VALS (Supp. Table 2) is a *M*=36 by *N*=16 matrix where each row-vector instance $d_{i,j}=DVALS(X_{i,j},y_{i+,j+})$ where *i*=year (2015 to 2017), *j*=month (January to December), $X_{i,j}$ is a 1 by *N*=15 row-vector containing all the variables for that unique year and month and $y_{i+,j+}$ is the malaria prevalence target value of the following unique month.

To exploit information from previous months, we encoded six regression tasks M***_m_*** (*m* = 1 to 6) that, while still predicting $y_{i+,j+}$ malaria prevalence of the following *i*=year *j*=month, add to that unique (*i, j*) month instance the 1 by *N* (N = number-of-variables = 15) row-instance variables of the previous (*m*-1) months where *m* = 1 to 6 (Table 4). The resulting dimensionality of (*i, j*) instance row-vectors for each M***_m_*** task are 1 by *N* = [(number-of-variables x *m*) + 1] = [(15 x *m*) + 1] (Table 4). This encoding creates the following tasks Tasks+1 M1 to M6 (DT1M1, DT1M2, DT1M3, DT1M4, DT1M5 and DT1M6) for both DTRAS and DVALS datasets (Table 4). Finally, the DTRAS and DVALS Task+1 datasets were further processed to remove the year (non-aggregated) column(s) variable (Supp. Table 2).

**Supervised Machine Learning Regression Approaches.**

To build the predictive regression system we used Generalized Linear Models (GLM), Ensemble Methods (EM) and Support Vector Machines (SVM) within a supervised learning framework (Figures 2 and 4).

**Generalized Linear Models (GLM):**

We used a set of GLM regression algorithms in which the target outcome $y$ (dependent variable) is expected to be a linear combination of the input features (independent variables). A GLM such as linear regression (LR)or ordinary least squares (OLS) fits a linear model with vector coefficients $w=(w_{1},...,w_{p})$, where *p*=number-of-dimensions, that minimizes the residual sum of squares between target outcome $y$ and the predicted outcome $\hat{y}$ of the lineal model where *X* is a *M* by *p* matrix with *X_i_*=1 to *M* row-vectors containing the features *p* of *i^th^*-example and *y* is a *M* by 1 column-vector containing *y_i_* target value of the *i^th^*-example.

*Ridge Regression (RR)*: RR imposes a L2-norm penalty on the regression coefficients. The ridge coefficients minimize a penalized sum of squares where $\alpha\geq0$ controls the amount of shrinkage.

*Least Absolute Shrinkage and Selection Operator (LASS0)*: The LASSO estimate solves the minimization of the least-squares penalty with a L1-norm prior regularization added. The optimization problem is where $\alpha$ is a constant that controls the degree of sparsity of the coefficients estimated and is the L1-norm of the parameter vector.

*Elastic Net (EN)*: The EN takes advantage of both RR (L2-norm) and LASSO (L1-norm) prior regularization. The combination has the capacity to learn sparse models with few non-zero weights such as in LASSO while maintaining regularization properties of RR. The convex combination of L1-norm and L2-norm is controlled by the L1-ratio parameter $\rho$. The optimization problem is:

The EN has useful properties where there are multiple features correlated with one another. While LASSO will pick one of these at random, the EN is more likely to pick the correlated features.

*Least Angle Regression (LARS)*: Similar to forward stepwise regression, at each step it finds the predictor most correlated with the response, but it continues in a direction that is equiangular between predictors when there are multiple equally correlated predictors^1^.

*LARS-LASSO*: Implements LASSO using the LARS algorithm^1^ instead of a coordinate descent.

**Ensemble Methods**

*Random Forests (RF)*:

RF is a type of bagging ensemble method whereby a set of decision trees are learned on randomly sampled subsets of features and training data points. As in bootstrap sampling, data samples are drawn with replacement, helping to ensure the base learners are de-correlated. This method aims to reduce variance of the estimator and decrease issues of over-fitting common to standard decision trees. Predictions are then made by averaging the predictions from the set of individual trees^2^. The number of trees in the forest can be tuned as a parameter, as well as parameters of the individual trees themselves. These include 1) the maximum number of features considered when splitting at a node; 2) the maximal depth a tree can be grown until; 3) the minimum number of data samples necessary for a split; 4) the minimum number of data samples at leaf nodes.

**Support Vector Machines**

*Support Vector Regression (SVR)*: belongs to the family of Support Vector Machine learning algorithms ^3^, adapted for predicting continuous outputs^4^. The problem can be described as fitting a function $f(x)$ to a set of training examples such that any deviations from the observed targets are less than $\varepsilon$ which defines a margin around predictions where deviations are not penalized. The aim is to maximize the margin for a lower generalization error. The final solution results in a subset of training points that define the margin, the so-called support vectors.

Considering a linear function $f(x)=\langle w,x\rangle+b$, the norm can be minimized

subject to $\{\begin{matrix} y_{i}-\langle w,x\rangle-b\leq\varepsilon+\xi^{+} \\ \langle w,x\rangle+b-y_{i}\leq\varepsilon+\xi^{-} \\ \xi^{+},\xi^{-}\geq0 \end{matrix}$

where slack variables $\xi^{+}$,$\xi^{-}$ are incorporated to allow for errors outside the $\varepsilon$ range and form a constrained optimization problem. A constant $C\geq0$ acts as a regularization parameter, controlling the bias-variance trade-off or the model fit.

We used a radial basis function kernel for the implicit mapping of features onto high or infinite dimensions where the parameter $\gamma>0$ is the Gaussian and thus can be used to control how much data points influence the function fit.

The optimal solution is $\hat{w}=\sum_{i=1}^{N} \alpha_{i}x_{i}$ so that future predictions can be made by

$$f(x)=\hat{b}+\sum_{i,j=1}^{N} a_{i}K(x_{i},x_{j})$$

**Error Measures and Parameter Tuning**

Mean Absolute Error (MAE) and Mean Square Error (MSE) measures were used when evaluating the quality of predictions of malaria prevalence.

MAE measures the average magnitude of the errors in a set of predictions without considering their direction. MAE is a risk metric corresponding to the expected value of the absolute error loss or L1-norm loss:

where N*_y_* is the number-of-years used for evaluation, $y_{i,j}$ is the true prevalence value of the month *j of the year* *i* and $\hat{y}_{i,j}$ is the corresponding predicted prevalence value. MAE is a scale dependent measure which is intuitive to interpret and its inclusion in malaria forecasting evaluation has been recommended ^5^.

MSE measures the average of the squares of the errors in a set of predictions incorporating both variance on the estimator and its bias. MSE is a risk metric corresponding to the expected value of the squared (quadratic) error or loss:

where *N* is the number-of-years used for evaluation, $y_{i,j}$ is the true prevalence value of the month *j of the year* *i-*and $\hat{y}_{i,j}$ is the corresponding predicted prevalence value. MSE is also a scale dependent measure and is a commonly used indicator of trained model performance within a study.

For LASSO-LARS-AIC alpha parametrization we used Akaike Information Criterion (AIC)^6^ and for LASSO-LARS-BIC the Bayesian Information Criterion (BIC)^7^ both used for model selection.

**Algorithm Parametrization, Evaluation and Model Selection.**

For algorithm parametrization and evaluation, each of the training DTRAS datasets encoding the DT1M1 to M6 regression tasks, DT1M1-DTRAS to DT1M6-DTRAS (Supp. Table 2), were randomly split 10^3^ times into a Train Set (TS) containing 75% of the instances and a Held-Out Test Set (HOTest) containing 25% of the instances (Figure 2). The TS is a *M* by *N* matrix where *M* = ceiling (0.75 x number-of-instances) and *N* = (number-of-variables per each T1M1 to M6 tasks) Table 4. The HOTest is a *M* by *N* matrix where *M* = (rest of the number-of-instances not in TS) and *N* = (number-of-variables per each T1M1 to M6 tasks).

Each TS [X, y] was then used for the parameterization of each regression task algorithm within the framework (Figure 2). For tuning the hyper-parameter alpha (regularization strength) for RR, a set of alphas = [1^-3^, 1^-2^, 1^-1^, 1, 10, 10^2^] were used and the best alpha selected by 5-fold cross-validation on the TS (Figure 2). For alpha selection in LASSO, EN, LARS, LASSO-LARS, we used model-specific iterative fitting along regularization path and selecting the best model by 5-fold cross-validation on the TS (Figure 2). Selection of best parameters was carried out using MSE as implemented in the scikit-learn Python library^8^.

For LASSO-LARS alpha parametrization we also used the Akaike Information Criterion (AIC) and the Bayesian Information Criterion (BIC) (Figure 2). These provide an optimal estimate of the regularization parameter by computing a single regularization path instead of several when k-fold cross-validation is used. The AIC and BIC criteria are useful for selecting the value of the regularization parameter by making a trade-off between the goodness of fit and the complexity of the model.

We parametrized the meta-estimator RF with number-of-trees=10; maximum-features = number-of-features; nodes are expanded until all leaves are pure or until all leaves contain less than 2; using bootstrap when building trees (Figure 2).

For SVR we used a Gaussian kernel and carried out 5-fold cross-validation to parametrize C and γ with the following grid search C = [1, 10, 10^2^, 10^3^, 10^4^] and γ = [1, 10^-1^, 10^-2^, 10^-3^, 10^-4^] respectively.

After each parametrization, the algorithm was trained on the TS with the optimal parameters and predictions were made on the target outcome $\hat{y}$ (prevalence of following month) on the X instances of HOTest (Figure 2). The trained algorithm test performance was then measured by MAE and MSE (Figure 2) and mean±SD of MAE and MSE over the 10^3^ random splits of DT1M1-DTRAS to DT1M6-DTRAS (Figure 3).

**L1-L2 Ratio and regularization strength Elastic Net Parametrization**

After selecting EN as the main ML algorithm for the system, we parametrized both $\alpha$ (regularization strength) and the L1-norm to L2-norm ratio (L1Ratio) as illustrated in Figure 4a . Each of the training DTRAS datasets encoding the DT1M1 to M6 regression tasks, DT1M1-DTRAS to DT1M6-DTRAS, were randomly split 10^3^ times into a Train Set (TS) containing 75% of the instances and a Held-Out Test Set (HOTest) containing 25% of the instances (Figure 4a). The TS is a *M* by *N* matrix where *M* = ceiling (0.75 x number-of-instances) and *N* = (number-of-variables per each T1M1 to M6 tasks). The HOTest is a *M* by *N* matrix where *M* = (rest of the number-of-instances not in TS) and *N* = (number-of-variables per each T1M1 to M6 tasks).

For EN $\alpha$ and L1Ratio we used model-specific iterative fitting along regularization path and selecting the best model by 5-fold cross-validation on the TS (Figure 4a). Selection of best parameters was carried out using MSE as implemented in the scikit-learn Python library^8^. After each parametrization, the EN was trained on the TS with the best parameters and predictions for target outcome $\hat{y}$ (prevalence of following month) were made on the X instances of HOTest (Figure 4a). The trained algorithm test performance was then measured by MAE and MSE (Figure 4a). The mean±SD of MAE, mean±SD of MSE, mean±SD of $\alpha$s and median±IQR of L1Ratio were plotted over the 10^3^ random splits of DT1M1-DTRAS to DT1M6-DTRAS (Figure 5). The $y_{i}$ (true prevalence value of all instances $i$) versus the mean of $\hat{y}_{i}$ (mean predicted prevalence value over the times the instance $i$ was included in the HOTest) is plotted in Figure 6 for all regression tasks.

**Software and Libraries**

We coded the framework using the Python programming language version 3.6.5 and with the open-source scikit-learn (version 0.19.1) machine-learning library^8^. Anaconda Navigator 1.8.4 was used to standardize Python environment (Python 3.6.5, Anaconda, Mar 29^th^, 2018). Dataset aggregates and statistical analysis were carried out with StataSE v12 and Python scripts; plots and figures were plotted with GraphPad and Miner3D.

**Fig. S1. REMPS evaluation of true *vs.* predicted prevalence values on Held-Out Test Set over 10^3^ random samplings of the training DTRAS dataset.**

**a.** to **f.** True prevalence value $y$ (dotted black line) vs. mean predicted prevalence value $\hat{y}$ (red line) for all the regression tasks DT1M1 to DT1M6 respectively over 10^3^ random samplings.

DTRAS=Ibadan Dataset Training Set [from 1996 to 2014].

**Fig. S2 REMPS (trained without parasite densities) predicted prevalence on validation set within regionally relevant tolerance-error.**

REMPS predicted prevalence for all validation years 2015, 2016, 2017 and all regression tasks DT1M1 to DT1M6 (orange, blue, red, purple, green, yellow filled squares respectively) plotted against the true prevalence value (black circles) and true prevalence value +0.1 to -0.05 tolerance-error (shaded grey area).**Fig. S3. Proposed REMPS Deployment Scenarios**

**a.**

**b.**

**a.** *Scenario 1*: a locally deployed REMPS is parametrized to provide accurate regional-specific estimates of next-month malaria prevalence. *Scenario 2*: a network of locally deployed REMPS systems push their regional predictions to a distributed ledger that ensures consistency, immutability and trust among participating nodes and provides access to relevant stakeholders.

**b.** A high-level view of the interaction of individual local REMPS where a distributed ledger provides an interface for third parties to access the fine-grained burden of disease data.

**Supplementary Table 1.** Previous Machine Learning Approaches

| **STUDY** | **OUTCOME** | **ML** | **VARIABLES** | **DATASET** | **MEASURE &**  **PERFORMANCE** |
| --- | --- | --- | --- | --- | --- |
| Kiang *et al*.  (2006) [7]  **Thailand** | Number of  Monthly  Malaria  Cases | NN-MLP | Rainfall  Temperature  Relative  Humidity  Vegetation Index | *Train:*  1994-1999  *Test Set*:  1 year left out | NRMSE  72% Train  62% Test |
| Zacarias *et al.*  (2013) [9]  **Mozambique** | Monthly  Malaria  Cases | RT  RF | IRS campaigns  Month  Min and Max Temperature  Rainfall  Temperature variance  Humidity  Administrative District | *Train*:  1999-2006  *Test*:  2007 | MSE >= 2x10^-2^  R^2^ [0.4 to 0.8] |
| Chintalapati *et al.*  (2014) [11]  **India** | Monthly  Malaria  Incidence | SVM-FFA  SVM  NN  ARMA | Rainfall  Temperature  Relative Humidity | *Train:*  1998-2001  *Test*:  2000 & 2002 | NMSE <=0.2  R^2^ [0.4 to 0.9] |
| Sharma *et al.*  (2015) [10]  **India** | Monthly  Malaria  Cases.  Outbreak (Y/N)  [classification] | SVM  NN | Rainfall  Temperature  Humidity | 2011-2014 | RMSE = 0.12  ROC = 0.89 |
| Buczak *et al.*  (2015) [9]  **South Korea** | Weekly  Malaria  Incidence  [classification] | FARM  DT  RF  SVM  Holt-Winters | No. of ITNs  Financial Aid  Distance to Demilitarized Zone  Elevation  Rainfall  Land Surface Temperature  Vegetation Indices  Southern Oscillation Index  Sea Surface | Train:  2004 to 2006,  2012  Test:  2007, 2013 | PPV  [0.6 to 0.8]  Sensitivity  [0.3 to 0.8] |
| **This Study**  (2019)  Ibadan  Metropolis  (3 million)  Second largest  Urban in Nigeria | Monthly  Malaria  Prevalence  [regression]  DT1M1  to DT1M6 | **EN**  LASSO  RR  RF  SVR | Monthly:  Total Number Children  Children Malaria Cases (prevalence)  Median & IQR Age malaria-negative  Median & IQR Age malaria-positive  Mean & STD Parasite-Density  Rainfall  Min, Max & Mean Temperature | *Train Set*  1996 to 2014  *Validation Set:*  2015  2016  2017 | MAE<=6x10^-2^  MSE <=7x10^-3^ |

**Supplementary Table 2.** Overview of clinical characteristics, aggregated by year, of training and validation cohorts.

|  | Year | Screened  N | F / M  (%) | MP (+ve)  N | MP (-ve)  N | Age MP (+ve)  months  median (IQR) | Age MP (-ve)  months  median (IQR) | MP Density  MPs/μl  log[mean (se)] |
| --- | --- | --- | --- | --- | --- | --- | --- | --- |
| TRAINING (DTRAS) | **1996** | 2,295 | 47 / 53 | 627 | 1,668 | 36 (42) | 24 (50) | 4.1 (3.0) |
|  | **1997** | 3,732 | 44 / 56 | 879 | 2,853 | 36 (55) | 24 (48) | 4.2 (2.8) |
|  | **1998** | 2,705 | 42 / 58 | 1,300 | 1,405 | 36 (44) | 27 (50) | 4.2 (3.0) |
|  | **1999** | 2,380 | 45 / 55 | 919 | 1,461 | 30 (43) | 29 (50) | 4.2 (3.0) |
|  | **2000** | 2,404 | 41 / 59 | 669 | 1,735 | 36 (43) | 24 (45) | 4.3 (3.2) |
|  | **2001** | 2,656 | 49 / 51 | 762 | 1,894 | 51 (58) | 50 (64) | 4.5 (3.2) |
|  | **2002** | 2,688 | 48 / 52 | 872 | 1,816 | 48 (55) | 48 (60) | 4.4 (3.1) |
|  | **2003** | 2,716 | 45 / 55 | 915 | 1,801 | 36 (43) | 34 (52) | 4.3 (3.0) |
|  | **2004** | 4,123 | 45 / 55 | 856 | 3,267 | 36 (42) | 24 (52) | 3.8 (2.9) |
|  | **2005** | 5,504 | 44 / 56 | 1,284 | 4,220 | 36 (45) | 24 (49) | 4.1 (2.9) |
|  | **2006** | 5,497 | 43 / 57 | 1,055 | 4,442 | 30 (45) | 26 (48) | 4.2 (3.0) |
|  | **2007** | 6,882 | 42 / 58 | 1,083 | 5,799 | 31 (43) | 24 (48) | 4.2 (2.9) |
|  | **2008** | 5,341 | 42 / 58 | 966 | 4,375 | 36 (42) | 27 (49) | 4.2 (2.9) |
|  | **2009** | 6,585 | 43 / 57 | 838 | 5,747 | 42 (54) | 28 (48) | 4.4 (3.1) |
|  | **2010** | 6,258 | 43 / 57 | 965 | 5,293 | 36 (43) | 21 (49) | 4.5 (3.2) |
|  | **2011** | 5,207 | 43 / 57 | 689 | 4,518 | 35 (54) | 28 (44) | 4.4 (3.2) |
|  | **2012** | 5,180 | 42 / 58 | 737 | 4,443 | 39 (54) | 22 (51) | 4.5 (3.3) |
|  | **2013** | 5,014 | 44 / 56 | 589 | 4,425 | 36 (45) | 23 (49) | 4.6 (3.4) |
|  | **2014** | 5,073 | 43 / 57 | 898 | 4,175 | 30 (50) | 24 (48) | 4.6 (3.3) |
|  | ***TOTAL*** | *82,240* | *44 / 56* | *16,903* | *65,337* | *36 (47)* | *28 (50)* | *4.3 (3.0)* |
|  | | | | | | | | |
| VALIDATION (DVASL) | **2015** | 3,091 | 49 / 51 | 551 | 2,540 | 40 (55) | 32 (49) | 4.6 (3.2) |
|  | **2016** | 3,743 | 47 / 53 | 534 | 3,209 | 41 (48) | 34 (51) | 4.5 (3.1) |
|  | **2017** | 3,737 | 48 / 52 | 641 | 3,096 | 48 (65) | 68 (62) | 4.5 (2.5) |
|  | ***TOTAL*** | *10,571* | *48 / 52* | *1,726* | *8,845* | *43 (56)* | *45 (54)* | *4.5 (2.9)* |

IQR= interquartile range; MP(+ve) = malaria parasite positive; MP(-ve) = malaria parasite negative; MP Density = malaria parasites per microliter.

**Supplementary Table 3.** Encoding of Regression Tasks

| **Dataset Task+1 M*_m_*-DTRAS** & **Dataset Task+1 M*_m_*-DVALS**  (DT1M*_m_*-DTRAS) & (DT1M*_m_*-DVALS)  where ***m*** = 1 to 6;  ***DTRAS i***=year (1996 to 2014); **DVALS *i***=year (2015 t0 2017); ***j***=month (Jan to Dec) | | | | | | | | |
| --- | --- | --- | --- | --- | --- | --- | --- | --- |
| Each row-vector **d** of DT1M*_m_*-DTRAS = $d_{m,DTRASi,j}=DTRAS(x_{m,DTRASi,j},y_{DTRASi,j})$ Each row-vector **d** of DT1M*_m_*-DVALS = $d_{m,DVALSi,j}=DVALS(x_{m,DVALSi,j},y_{DVALSi,j})$ | | | | | | | | |
| Target $y_{DTRASi,j}$ = malaria prevalence of ***DTRAS i, j*** following month  Target $y_{DVALSi,j}$ = malaria prevalence of ***DVALS i, j*** following month | | | | | | | | |
| $x_{m,i,j}$= append ***i, j*** previous (***m***-1) months = [1 by *N*=(15 x *m*) row-vector] | | | | | | | | |
| **DTask+1 M*_m_*** | **M6** | **M5** | **M4** | **M3** | **M2** | **M1** | **d_all_** | **d_final_**  **_remove year column(s)_** |
| Task+1 M*_m_*=1 (DT1M1) | NO | NO | NO | NO | NO | YES | 1 by 16 | 1 by 15 |
| Task+1 M*_m_*=2 (DT1M2) | NO | NO | NO | NO | YES | YES | 1 by 31 | 1 by 29 |
| Task+1 M*_m_*=3 (DT1M3) | NO | NO | NO | YES | YES | YES | 1 by 46 | 1 by 43 |
| Task+1 M*_m_*=4 (DT1M4) | NO | NO | YES | YES | YES | YES | 1 by 61 | 1 by 57 |
| Task+1 M*_m_*=5 (DT1M5) | NO | YES | YES | YES | YES | YES | 1 by 76 | 1 by 71 |
| Task+1 M*_m_*=6 (DT1M6) | YES | YES | YES | YES | YES | YES | 1 by 91 | 1 by 85 |

Example of an encoded row-vector *see Table 3 for variable index and names

| **Example of (index *x*)* features of a 1 by 15 d_final_ row-vector of DT1M1 Tasks** | | | | | | | | | | | | | | |
| --- | --- | --- | --- | --- | --- | --- | --- | --- | --- | --- | --- | --- | --- | --- |
| **2** | **3** | **4** | **5** | **6** | **7** | **8** | **9** | **10** | **11** | **12** | **13** | **14** | **15** | $y$ | |
| features | | | | | | | | | | | | | | target | |

**SI References**

1. Efron, B. *et al.* Least angle regression. *Ann. Stat.* **32**, 407–499 (2004).

2. Breiman, L. Random forests. *Mach. Learn.* (2001) doi:10.1023/A:1010933404324.

3. Cristianini, N. & Shawe-Taylor, J. *An introduction to Support Vector Machines*. *Cambridge University Press* (2000). doi:10.1017/S0263574700232827.

4. Drucker, H., Burges, C., Kaufman, L., Smola, A. & Vapnik, V. Support Vector Regression Machines. *Adv. Neural Inf. Process. Syst.* **9**, 155–161 (1996).

5. Zinszer, K. *et al.* A scoping review of malaria forecasting: past work and future directions. *BMJ Open* **2**, (2012).

6. Akaike, H. A New Look at the Statistical Model Identification. *IEEE Trans. Automat. Contr.* **19**, 716–723 (1974).

7. Schwarz, G. Estimating the Dimension of a Model. *Ann. Stat.* **5**, 461–464 (1978).

8. Pedregosa, F. *et al.* Scikit-learn: Machine Learning in Python. *J. Mach. Learn. Res.* **12**, 2825–2830 (2011).
